# Supplementary material for: The Relationship between Flow Experience and Burnout Symptoms: A Systematic Review
Source: Int J Environ Res Public Health. 2022 Mar 24;19(7):3865. doi: 10.3390/ijerph19073865 (PMC8998023; doi:10.3390/ijerph19073865)
Supplement: Supplementary file 1 [file ijerph-19-03865-s001.zip › ijerph-1581388-supplementary.pdf]

Table S1. Rating using an adapted version of the Newcastle-Ottawa Quality assessment scale (adapted from Rotenstein et al. [8]).

Study	Representativeness of the Sample	Sample Size	Ascertainment of Burnout	Ascertainment of Flow	Quality of Descriptive Statistics Reporting	Sum
Bakker & Geurts, 2004 [77]	1	1	1	0	1	4
Baumgarten et al., 2020 [80]	1	0	1	1	0	3
Demerouti et al., 2012 [82]	1	0	1	1	1	4
Kasa & Hassan, 2015 [84]	1	0	1	1	0	3
Kasa & Hassan, 2016 [86]	1	1	1	1	0	4
Kasa & Hassan, 2019 [87]	1	0	1	1	0	3
Lavigne et al., 2012 [89] (Study 1)	0	0	1	1	1	3
Lavigne et al., 2012 [89] (Study 2)	1	1	1	1	1	5
Ljubin-Golub et al., 2020 [93]	0.5	0	1	1	1	3.5
Mäkikangas et al., 2010 [95]	0.5	1	1	1	1	4.5
Martínez-Zaragoza et al., 2014 [96]	1	0	1	1	1	4
Martínez-Zaragoza et al., 2017 [98]	1	0	1	1	1	4
Mosing et al., 2018 [99]	1	1	1	1	0.5	4.5
Rodríguez-Sánchez et al., 2011 [101]	1	0	0.5	0	1	2.5
Schiefele et al., 2013 [103]	1	0	1	1	1	4
Xanthopoulou et al., 2018 [107]	1	0	1	1	1	4
Xie et al., 2019 [108]	1	1	1	1	1	5
Zito et al., 2016 [109]	0	0	1	1	1	3

1) Representativeness of the sample
   1 point: Population contained multiple specialties and/or multiple institutions
   0 points: Population contained a single specialty and a single institution
2) Sample size:
   1 point: Sample size was ≥300 participants
   0 points: Sample size was <300 participants
3) Ascertainment of burnout:
   1 point: Well described and/or validated measurement tool, e.g., the MBI
   0 points: Poorly described measurement tool of uncertain validity or non-validated
   single-question screening tool
4) Ascertainment of flow:

1 point: Well described and/or validated measurement tool, e.g., the WOLF
0 points: Poorly described measurement tool of uncertain validity or non-validated
single-question screening tool or tools assessing another construct
5) Quality of descriptive statistics reporting:
1 point: Reported descriptive statistics to describe the population (e.g., age, sex)
with proper measures of dispersion (e.g., mean, standard deviation)
0 points: Descriptive statistics were not reported, were incomplete, or did not
include proper measures of dispersion

Note: This scale assesses quality in several domains: sample representativeness and size, ascertainment of burnout and flow, and statistical reporting quality.

Table S2. Additional multifactorial results.

Source	Multifactorial Results
Demerouti et al., 2012 [82]	**Multilevel estimates**<br>• EE at work (Y)<br>○ AB (X): Estimate = −0.09<br>○ EN (X): Estimate = −0.19 *<br>○ IM (X): Estimate = −0.08<br>○ AB*recovery (X): −0.12<br>○ EN*recovery (X): 0.34 ***<br>○ IM*recovery (X): 0.09<br>• EE at bedtime (Y)<br>○ AB (X): Estimate = −0.04<br>○ EN (X): Estimate = −0.16 *<br>○ IM (X): Estimate = −0.13<br>○ AB *detachment (X) = 0.09<br>○ EN *detachment (X) = −0.26 *<br>○ IM *detachment (X) = 0.14
Kasa & Hassan, 2015[a] [84]	**Mediation**<br>burnout symptoms (X) → flow (M) → OCB (Y): n.s
Kasa & Hassan, 2016 [86]	**Moderation**<br>burnout symptoms (X) and socio-cultural factor (MO) → flow (Y): n.s.
Kasa & Hassan, 2019[a] [87]	**Mediation**<br>burnout symptoms (X) → flow (M) → work-family conflict (Y): 0.08, −0.12: n.s.
Lavigne et al., 2012 [89] (Study 1)	**Path analysis**<br>• flow (Y)<br>○ Harmonious passion (X): $\gamma = 0.58$ ***<br>○ Obsessive passion (X): $\beta = -0.00$<br>• flow (X)<br>○ EE (Y): $\beta = -0.09$<br>○ CY (Y): $\beta = -0.43$ ***<br>○ RPA (Y): $\beta = -0.54$ ***<br>**Mediation *(Sobel Test z-Values)***<br>Harmonious passion (X) → flow (M) → EE (Y): −0.89<br>Harmonious passion (X) → flow (M) → CY (Y): 3.79 ***<br>Harmonious passion (X) → flow (M) → RPA (Y): 4.84 ***
Lavigne et al., 2012 [89] (Study 2)	**Cross-lag panel model**<br>• flow at T2 (Y)<br>○ Harmonious passion at T1 (X): $\gamma = 0.32$ ***<br>○ Obsessive passion at T1 (X): $\gamma = 0.09$<br>○ flow at T1 (X): $\gamma = 0.50$ ***<br>• flow at T2 (X)<br>○ EE at T2 (Y): $\beta = -0.31$ ***

	○ CY at T2 (Y): β = −0.34 ***<br>○ RPA at T2 (Y): β = −0.55 ***<br>flow at T1 did not predict burnout symptoms at T2<br>**Mediation *(Sobel test z-values)***<br>Harmonious passion at T1 (X) → flow at T2 (M) → EE at T2 (Y): −3.84 ***<br>Harmonious passion at T1 (X) → flow at T2 (M) → CY at T2 (Y): −4.16 ***<br>Harmonious passion at T1 (X) → flow at T2 (M) → RPA at T2 (Y): −4.53 ***<br>Obsessive passion at T1 (X) → flow at T2 (M) → EE at T2 (Y): −1.30<br>Obsessive passion at T1 (X) → flow at T2 (M) → CY at T2 (Y): −1.29<br>Obsessive passion at T1 (X) → flow at T2 (M) → RPA at T2 (Y): −1.32
Ljubin-Golub et al., 2020 [93]	**Mediation**<br>Autonomous motivation (X) → flow (M) → burnout symptoms (Y)<br>Indirect effect: β = −0.44 ***<br>Teachers' autonomy support (X) → autonomous motivation (M1) → flow (M2) → burnout symptoms (Y)<br>Indirect effect: β = −0.31 ***<br>flow (M2) → burnout symptoms (Y): −0.59 ***
Mäkikangas et al., 2010 [95]	**Moderation**<br>Job resources (X) and EE (MO) → flow (Y): n.s.<br>**Latent growth curve model**<br>Initial level of EE → initial level of flow: β = −0.31 *** and initial level for job resources: β = −0.39 ***<br>Initial level of EE → latent change factors of flow and job resources: n.s.
Martínez-Zaragoza et al., 2017 [98]	**Structural and measurement model** [b]<br>Associations between flow, health, burnout symptoms and approach coping are described<br>Flow as antecedent model: flow (X) → RPA (Y) = −0.16 **
Mosing et al. (2018) [99]	**Genetic and environmental influences**<br>r_g (flow proneness; emotional exhaustion) = −0.58 (genetic correlation)<br>r_e(flow proneness; emotional exhaustion) = −0.23 (environmental correlation)<br>*r*(flow proneness; emotional exhaustion) = −0.23 (controlled for shared genetic and familial factors) ***
Schiefele et al. (2013) [103]	**Structural equation model** [b]<br>Exogenous variables: self-efficacy, didactic interest, educational interest and subject interest<br>Outcomes: EE, RPA, CY, enjoyment and flow<br>• flow (Y)<br>○ EE (Y): n.s.<br>○ CY (Y) = n.s<br>○ RPA (Y) = n.s.
Xanthopoulou et al., 2018 [107]	**Multilevel path analysis**<br>• flow (M)<br>○ deep acting (X)= 0.13 **<br>○ surface acting (X) = −0.09 *<br>○ need for recovery (Y) = −0.25 **<br>• EE (M)<br>○ deep acting (X)= −0.13 *<br>○ surface acting (X) = 0.24 ***<br>○ need for recovery (Y): 0.45 ***<br>**flow & EE: Unstandardized estimates = −0.07 *** **
Zito et al., 2016 [109]	**Mediation**<br>• flow (M)<br>○ Job resources (X): 0.49<br>○ Job demands (X): −0.27<br>○ EX (Y): −0.70<br>Indirect effects:<br>Job resources (X)→ flow (M) → EX (Y): Estimate = −0.34 ***<br>Job demands (X)→ flow (M) → EX (Y): Estimate = 0.19 ***

Note. EE = emotional exhaustion, CY = cynicism, RPA = reduced personal accomplishment, EX = exhaustion, AB = absorption, EN = enjoyment, IM = intrinsic motivation, T = timepoint, X = independent variable, M = mediator, MO = Moderator, Y = dependent variable, OCB = organizational citizenship behavior, n.s. = not significant (statistical parameters not given). [a] The studies may have been based on the same data. [b] Due to the large number of factors considered in the models, a presentation of all interrelationships has been omitted. * $p < 0.05$; ** $p < 0.01$; *** $p < 0.001$. For reasons of clarity and comprehensibility, the labels of the various subscales were harmonized and reversed recoded where appropriate.
